# Selected Plant Extracts Regulating the Inflammatory Immune Response and Oxidative Stress: Focus on *Quercus robur*

**DOI:** 10.3390/nu17030510

**Published:** 2025-01-30

**Authors:** Rawan Nehme, Arthur Chervet, Caroline Decombat, Ola Habanjar, Lucie Longechamp, Amandine Rousset, Pierre Chalard, Mael Gainche, Francois Senejoux, Didier Fraisse, Edith Filaire, Jean-Yves Berthon, Mona Diab-Assaf, Laetitia Delort, Florence Caldefie-Chezet

**Affiliations:** 1 Université Clermont-Auvergne, INRAE, UNH, Unité de Nutrition Humaine, CRNH-Auvergne, 63000 Clermont-Ferrand, France; rawan.nehme@doctorant.uca.fr (R.N.); arthur.chervet@doctorant.uca.fr (A.C.); caroline.decombat@uca.fr (C.D.); ola.habanjar@uca.fr (O.H.); lucie.longechamp@uca.fr (L.L.); francois.senejoux@univ-fcomte.fr (F.S.); didier.fraisse@uca.fr (D.F.); edithfilaire@gmail.com (E.F.); laetitia.delort@uca.fr (L.D.); 2 Greentech, Biopôle Clermont-Limagne, 63360 Saint-Beauzire, France; amandinerousset@greentech.fr (A.R.); jeanyvesberthon@greentech.fr (J.-Y.B.); 3 Institut de Chimie de Clermont-Ferrand, Université Clermont Auvergne, Clermont Auvergne INP, CNRS, 63000 Clermont-Ferrand, France; pierre.chalard@sigma-clermont.fr (P.C.); mael.gainche@sigma-clermont.fr (M.G.); 4 Faculty of Sciences II, Lebanese University Fanar, Beirut 1500, Lebanon; mdiabassaf@ul.edu.lb

**Keywords:** inflammation, antioxidant, plant extracts, *Quercus robur*, macrophages

## Abstract

**Background/Objectives:** Inflammation is a vital response of the immune system, frequently linked to the development and progression of numerous chronic and autoimmune diseases. Targeting inflammation represents an attractive strategy to prevent and treat these pathologies. In this context, many pathways, including pro-inflammatory cytokines secretion, NFκB activation, reactive oxygen species (ROS) production, inflammasome activation and arachidonic acid metabolism could be highlighted and addressed. Several plant materials have traditionally been used as effective and non-harmful anti-inflammatory agents. However, well-established scientific evidence is lacking, and their mechanisms of action remain unclear. The current article compares the effects of seven plant extracts, including *Quercus robur* L. (Oak), *Plantago lanceolata* L. (narrowleaf plantain), *Plantago major* L. (broadleaf plantain), *Helichrysum stoechas* L. (immortelle or helichrysum), *Leontopodium nivale alpinum* Cass. (edelweiss), *Medicago sativa* L. (alfafa) and *Capsella bursa-pastoris* Moench (shepherd’s purse) on different inflammatory pathways. **Results**: All of the plant extracts significantly affected ROS production, but their action on cytokine production was more variable. As the *Quercus robur* extract showed the highest efficacy in our models, it was subsequently assessed on several inflammatory signaling pathways. *Quercus robur* significantly decreased the secretion of IFNγ, IL-17a, IL-12, IL-2, IL-1β and IL-23 in stimulated human leucocytes, and the expression of *TNFα*, *IL-6*, *IL-8*, *IL-1β* and *CXCL10* in M1-like macrophages. Additionally, a significant reduction in PGE2 secretion, COX2, NLRP3, caspase1 and STAT3 expression and NFκB p65 phosphorylation was observed. **Conclusions**: Our results clearly indicate that *Quercus robur* has a potent anti-inflammatory effect, making it a promising candidate for both the treatment and prevention of inflammation and related diseases, thereby promoting overall well-being.

## 1. Introduction

The body’s immune system’s basic reaction to microbial invasion or pathogens, tissue damage or damaged cells, irritants or other harmful stimuli is inflammation. It is orchestrated by a complicated interaction between immune cells, cytokines, chemokines and inflammatory mediators [1]. Innate and adaptive immune responses are involved in this highly regulated and dynamic biological process, which usually has four interconnected phases [2]: (1) The initiation phase, where immune cells, such as dendritic cells and macrophages, identify the damaging stimulus and trigger the production of inflammatory mediators, such as cytokines and chemokines. Further immune cells are then recruited to the affected area by these mediators [3]. (2) Vasodilation and enhanced vascular permeability at the site to allow immune cells, plasma proteins and fluid to enter the affected tissue, leading to classical symptoms of inflammation, i.e., redness, swelling, pain and heat. (3) Immune cells, especially neutrophils and monocytes, are then recruited to the site of inflammation by chemotaxis along a chemical signal gradient. Neutrophils are the first responders and are essential in defending against the initial insult, while monocytes differentiate into macrophages, which carry out phagocytosis to eliminate debris and pathogens. (4) Pro-resolution factors are released to aid in the resolution of inflammation once the harmful agent has been removed, allowing for tissue healing. Immune cells that are essential for stopping the inflammatory response and starting tissue regeneration and repair include regulatory T cells and macrophages [4]. This acute immune response is tightly regulated and serves as a protective mechanism to maintain tissue homeostasis and restore normal functioning [5]. However, an abundance of new evidence shows that persistent or dysregulated inflammation can lead to the development and progression of chronic pathologies including cardiovascular diseases, metabolic disorders, neurodegenerative conditions, cancers and inflammatory bowel disease [6].

A complex network of signaling pathways is essential for orchestrating the body’s immune response to various stimuli. Among them, Nuclear Factor-Kappa B (NFκB), a transcription factor, plays a central role in regulating the expression of pro-inflammatory genes, including cytokines such as tumor necrosis factor alpha (TNFα) and interleukin-6 (IL-6), chemokines and other inflammatory mediators [7]. Another important pathway is the NLRP3 (Nucleotide-binding domain, leucine-rich repeat-containing protein 3) inflammasome pathway, which, when activated, leads to the release of pro-inflammatory cytokines IL-1β and IL-18, amplifying the inflammatory response [8]. Dysregulation of these and others pathways can contribute to chronic inflammation inducing various inflammatory diseases.

Oxidative stress, defined as the imbalance between the production of reactive oxygen species (ROS) and the body’s ability to detoxify and neutralize them, is closely linked to inflammation and their relationship is crucial to understand the immune response and various inflammatory processes. The connection between them has been clearly documented and has different aspects, among them: (1) ROS are involved in activating NFκB and Mitogen-activated protein kinases (MAPKs), leading to the production of pro-inflammatory cytokines and chemokines; (2) Oxidative stress can damage cellular components such as DNA, proteins and lipids, leading to inflammation; (3) ROS can activate inflammasomes leading to the maturation and secretion of IL-1β and IL-18, which further promote inflammation; (4) In chronic inflammation, the persistent activation of immune cells, such as macrophages and neutrophils, leads to an ongoing ROS production and a sustained oxidative stress, contributing to tissue damage and maintenance of inflammation [9].

Depending on the underlying cause and severity of inflammation, numerous effective therapies may be used. Aspirin and ibuprofen are two examples of non-steroidal anti-inflammatory medicines (NSAIDs), which are among the most commonly prescribed treatments for the treatment of inflammation worldwide. They reduce inflammation and alleviate pain by inhibiting the production of prostaglandins, key mediators implicated in the inflammatory response [10]. In more severe cases of inflammation, prednisone and dexamethasone, corticosteroids that act by suppressing the immune response, can be prescribed [11]. Well-known adverse events are associated with the use of these two anti-inflammatory groups, from gastro-intestinal toxicity to cardiovascular problems, and such complications may decrease the quality of life of patients and can require high-cost management. In addition, several ‘targeted’ biologic agents such as TNF inhibitors, IL inhibitors and monoclonal antibodies are used in the treatment of chronic inflammatory conditions and autoimmune diseases [12]. However, despite key advances and remarkable progress in the management of chronic inflammation, this disease is not entirely curable and the identification of efficient and well-tolerated molecules that can treat inflammation is of greater importance.

On the other hand, the use of plant-based treatments, commonly referred to as traditional medicine, is also widespread and growing globally. Plants offer a valuable source of bioactive compounds and a wide variety of chemical structures that could be used as potential therapeutic candidates. Several plant extracts have been used to treat inflammation and have gained popularity due to their safety and potential benefits [13]. However, scientific evidence may be limited or inconclusive for some of them and additional studies are required to elucidate their mechanisms of action and potential targets in order to find a new generation of anti-inflammatory agents.

Here, we investigated the antioxidant and anti-inflammatory effects of seven plant-based extracts selected for their therapeutic potential, with the aim of exploring their efficacy in the field of inflammation: *Quercus robur* L. (Oak), *Plantago lanceolata* L. (narrowleaf plantain), *Plantago major* L. (broadleaf plantain), *Helichrysum stoechas* L. (immortelle or helichrysum), *Leontopodium nivale alpinum* Cass. (edelweiss), *Medicago sativa* L. (alfafa) and *Capsella bursa-pastoris* Moench (shepherd’s purse). These plants were chosen due to their well-established therapeutic actions and have long been used, frequently without scientific validation or a mechanistic understanding, to treat a variety of health conditions, such as oxidative stress, inflammation, respiratory and digestive disorders and skin diseases. Numerous bioactive compounds, including polyphenols, flavonoids and tannins, are identified in these plants and are thought to play a role in their medicinal benefits. Although being from different families and geographical areas, the seven plants share a common usage in herbal medicine to promote health and treat a variety of diseases.

## 2. Materials and Methods

### 2.1. Chemicals and Reagents

The solvents used in this study (water, ethanol, acetonitrile) as well as the chemicals (sodium acetate, acetic acid 100%, hydrochloric acid 37%, phosphoric acid, 2,4,6-Tris(2-pyridyl)-s-triazine (TPTZ), Trolox, Ferric chloride hexahydrate) were purchased from VWR (Radnor, PA, USA) at HPLC grade. All standards (primary reference standard grade) were acquired from Merck (Darmstadt, Germany).

### 2.2. Preparation of Plant Extracts

The seven plants used in this work: *Quercus robur* (bark), *Plantago lanceolata* (dry leaves), *Plantago major* (dry leaves), *Helichrysum stoechas* (dry flowers), *Leontopodium nivale alpinum* (dry aerial parts), *Medicago sativa* (dry aerial parts) and *Capsella bursa-pastoris* (dry aerial parts) were purchased from European suppliers. Each plant was grinded into fragments ranging from 0.1 to 1 cm except the bark of pedunculate oak (*Quercus robur*) which was dried and shredded into pieces between 0.1 and 2 cm. For each sample, 100 g were extracted using a 2000 mL ethanol–water mixture (50%, *v*/*v*) and stirred on a rotary shaker at 120 rpm for 4 h at room temperature. The mixture was then filtered through filter paper to separate the solid residue from the extract. The resulting filtrate was concentrated using a rotary evaporator (at 40 °C) yielding a powder representing the crude extract.

### 2.3. Quantification of Molecules of Interest by HPLC-UV

Potentially active molecules in the plant extracts were analyzed using high-performance liquid chromatography (HPLC) equipped with a diode array detector (190–800 nm) from Agilent (Santa Clara, CA, USA). These molecules were identified through a bibliographic study of the phytochemistry of each plant. Then, commercial standards were used to detect and quantify some molecules in each extract as listed in Table 1.

### 2.4. Determination of Antioxidant Activity by Ferric-Reducing Antioxidant Power (FRAP) Assay

The FRAP reagent solution was obtained by combining acetate buffer (20 mM, pH 3.6), TPTZ solution (10 mM in 40 mM HCl) and FeCl_3_·6H_2_O solution (20 mM) in a 10:1:1 *v*/*v*/*v* ratio. Trolox standard solutions were prepared with concentrations ranging from 0.1 to 1 mg/L. To assess the antioxidant capacity, 50 μL of each plant extract or Trolox standard solution was added to 200 μL of the prepared FRAP reagent solution. The mixture was then incubated for one hour at 37 °C. Absorbance was measured at 593 nm and the FRAP values were calculated and expressed as μmol Trolox equivalents per gram of dry weight (μmol TE/g dw), indicating the antioxidant potential of the samples.

### 2.5. Blood Cells and Cell Culture

*Blood Leucocyte Preparation:* Blood samples were obtained from healthy human volunteers (n = 21) through the ‘Etablissement Français du Sang’, EFS, in Clermont-Ferrand, France. All donors provided written informed consent for the use of blood samples for research purposes in compliance with the EFS contract n°16-21-62 and the relevant articles of the French Public Health Code (L1222-1, L1222-8, L1243-4 and R1243-61). The blood samples were mixed with the ammonium chloride solution, and gentle agitation was performed for a short period not exceeding 5 min at room temperature. During this time, the red blood cells lyse, releasing hemoglobin and other cellular contents into the solution. After hemolysis, the sample was centrifuged to separate the leukocytes from the plasma and other cell debris. The leukocytes, remaining at the pellet, were then suspended in Roswell Park Memorial Institute medium-1640 (RPMI-1640, Gibco, ThermoFisher Scientific, Waltham, MA, USA) supplemented with fetal bovin serum (FBS, 10%) (Eurobio Scientific, Les Ulis, Île-de-France, France), gentamicin (50 μg/mL) and glutamine (Gln, 2 mM) (ThermoFisher Scientific, Waltham, MA, USA).

PBMC Preparation: Blood buffy coats were obtained from healthy human volunteers (n = 36) and layered on a simple gradient of Ficoll–Histopaque 1077^®^ (Sigma Aldrich, St. Louis, MO, USA). The top plasma layer was eliminated after centrifugation (400 g, 40 min at 20 °C), leaving a layer of monocytes and lymphocytes (peripheral blood mononuclear cells PBMCs) just above the 1.077 g/mL phase. Following two centrifugations (5 min, 400 g) and a wash with RPMI, the PBMCs layer was resuspended in 5 mL of supplemented RPMI (FBS 10%, gentamicin 50 μg/mL, and Gln 2 mM). For subsequent assays, the final cell concentration was adjusted to 10^6^ cells/mL.

The human monocytic leukemia cells: THP-1 cells (American Type Culture Collection ATCC, Manassas, VA, USA) were cultured in complete RPMI-1640 medium (10% FBS, 2 mM Gln and 50 µg/mL gentamicin). To differentiate the monocytes into macrophages, 4 × 10^5^ cells per mL were seeded in 6-well plates and incubated for three days in a complete growth medium containing 16.2 nM phorbol 12-myristate 13-acetate (PMA, Sigma-Aldrich, St. Louis, MO, USA) as previously described [14]. Following activation, macrophages were incubated for 24 h with 20 ng/mL interferon-gamma (IFN-γ, Gibco ThermoFisher Scientific, Waltham, MA, USA) and 10 pg/mL lipopolysaccharides (LPS, Sigma-Aldrich, St. Louis, MO, USA) to polarize them into M1-like macrophages.

### 2.6. Kinetics of ROS Production by Blood Leukocytes

The blood leucocytes were distributed into 96-well plates (n = 3 by plant extract) at the concentration of 10^6^ cells/mL. The cells were incubated with plant extracts at concentrations of 0, 10, 25, 50 or 100 µg/mL, along with and dihydrorhodamine 123 (DHR 123, 1 μM, Sigma-Aldrich), and were stimulated or not with 1 µM PMA for 120 min [15,16]. The oxidation of DHR 123 by ROS results in the production of rhodamine 123, which could be detected by measuring its fluorescence. Fluorescence readings were taken every 5 min over a 120-min period, with excitation at 485 nm and emission at 538 nm, using a Tecan Spark^®^ microplate fluorometer (Tecan Group, Männedorf, Zurich, Switzerland).

### 2.7. Leukocyte Viability

In parallel to ROS production measurement, the same blood leucocyte preparation was seeded in 96-well plates and incubated with the plant extracts at concentrations of 10, 25, 50 or 100 µg/mL, along with 0 or 1 µM PMA and 25 µg/mL of resazurin. Fluorescence intensity, with excitation at 544 nm and emission at 590 nm, was recorded at 30-min intervals for 2 h using a Fluoroskan^®^ microplate fluorometer (Ascent FL, ThermoFisher Scientific, Waltham, MA, USA).

### 2.8. Determination of Cytokine Concentrations

The production of ten human cytokines (IFNγ, IL-12 p70, IL-1β, IL-2, IL-23, IL-6, IL-8, IL-17a, MIP-1α and TNFα) was measured using ProcartaPlex™ Immunoassays (Thermo Fisher Scientific, Waltham, MA, USA). For that, PBMCs were seeded at 10^6^ cells/mL and incubated with or without phytohemagglutinin (PHA, 5 µg/mL, Sigma-Aldrich, St. Louis, MO, USA) [17] and plant extracts (0 or 50 µg/mL) for 24 h. All assays were performed in triplicate (n = 3 per extract). Using the manufacturer’s recommended ideal doses of standards and antibodies, cytokine concentrations were calculated. The plates were read in the Luminex Bio-Plex 200 System (Biorad, Marnes-la-Coquette, Île-de-France, France) and BioPlex Manager™ 4.1 software was used to evaluate the data using a five-parameter logistic regression (5PL) curve fitting.

### 2.9. Real-Time Quantitative PCR (RT-qPCR)

To evaluate gene expression, total RNA was extracted with TRIZOL reagent (Invitrogen, Thermo Fisher Scientific, Carlsbad, CA, USA). The RNA quantity and purity were assessed with a Tecan Spark^®^ system. DNase treatment was then performed to eliminate any residual genomic DNA (DNase I Amplification grade, Invitrogen, Waltham, MA, USA), followed by reverse transcription of RNA into cDNA using the HighCap cDNA RT Kit (RNase inhibitor, Invitrogen, Waltham, MA, USA) as per the manufacturer’s instructions. The cDNA samples were then diluted to 5 ng/μL. PCR Amplification was carried out using SYBRGreen PCR Master Mix (Applied Biosystem, Foster City, CA, USA) and primers (Table 2) on a StepOneTM instrument (Applied biosystem, Waltham, MA, USA). The experiments were carried out in duplicate for each data point. The reference gene β-actin was used as an internal control for the normalization of RNA quantity and quality differences among the samples. The relative quantification method (RQ = 2^−ΔΔCT^) was used to calculate the relative gene expression of given samples with ΔΔCT = [ΔCT (sample1) − ΔCT (sample2)] and ΔCT = [CT (target gene) – CT (reference gene)].

### 2.10. Flow Cytometer Assay

A BDTM LSR II flow cytometer was used to analyze the phenotype of the macrophages. For that, THP1 cells were differentiated, polarized and collected using 8 mM DPBS/EDTA. Cells were then adjusted to a density of 10^6^ cells/mL with DPBS and 1 mL of cell suspensions was aliquoted into polystyrene flow cytometry tubes for unstained controls, Fluorescence Minus One (FMO) controls and fully stained samples. CD14-PE-VIO 770-HUMAN (130-110-521, Miltenyi Biotec, Bergisch Gladbach, Germany) was used to identify M0 and CD80-PE-HUMAN-REA661 (130-123-253, Miltenyi Biotec, Bergisch Gladbach, Germany) to identify M1-like macrophages. For viability staining adjustments, ethanol-treated killed cells were used as controls and stained with Viobility Dye 405/520 (130-130-404) at the recommended dilution by the manufacturer. Cells were first stained with Viobility Dye for 10 min at 4 °C. Then, cells were stained with 4 µL CD14 and 4 µL CD80 in a total volume of approximately 100 μL as previously described [18]. After 30 min, cells were washed once with DPBS, centrifuged at 300 g for 5 min, resuspended in 350 µL of DPBS and then placed at 4 °C for acquisition. At least 30,000 live cells were counted to assess macrophage activation (M0%) and polarization (M1%). The results were analyzed using FACSDiva version 9.1 software (BD Biosciences) and are presented as the percentage of positive cells.

### 2.11. Measurement of Total and Phospho-NFκB p65 Level Using ELISA Immunoassay

Using the ELISA (NFκB p65 (Total/Phospho) Human InstantOne™ ELISA kit, Thermo Fisher Scientific, Waltham, MA, USA), the expression of NFκB p65 in the PBMCs was evaluated to investigate the impact of *Quercus robur* on the NFκB pathway [19]. The adjusted PBMCs solution was seeded in 96-well plates, stimulated or not with LPS (10 µg/mL) and incubated for 2 h in the presence or absence of *Quercus robur* extract (50 µg/mL). Pellets were collected for measurement of NFκB phosphorylation that was strictly performed according to the manufacturers’ protocol. The pre-coated ELISA plate was filled with total cell lysate, negative control and positive control. Then, total-NFκB p65 and phos-pho-NFκB p65 antibodies were separately incubated for an hour in each well. The detection reagent was then added to the wells, incubated to detect the captured total-/phospho-NFκB p65 protein and subsequently inhibited by the stop solution. The optical density (OD) of the yellow-colored product was determined at 450 nm. Next, the ratio of phospho-NFκB p65 to total-NFκB p65 ratio was computed. Data were compared to the positive control group (cells treated with LPS without extract) and expressed as a percentage of the control. All of the samples were tested in duplicate.

### 2.12. Statistical Analysis

Every experiment was carried out 3–6 times, and the results are shown as mean ± SEM. GraphPad Prism software version 8.0.1 (GraphPad Software, San Diego, CA, USA) was used for statistical analysis, with one-way ANOVA followed by Dunnett’s *t*-test for comparisons among multiple groups, and Student’s *t*-test for comparisons between two groups. *p*-values were calculated, with values <0.05, <0.01, <0.001, and <0.0001 (*, **, ***, ****, respectively) considered statistically significant.

## 3. Results

### 3.1. Extract Content and Antioxidant Capacity

Table 3 provides a detailed analysis of the extracts’ main constituents. FRAP experiments were used to assess the extracts’ potential to scavenge radicals. FRAP values, reflecting the direct antioxidant activity of the extracts, were found as follows (Table 4): for *Quercus robur*, 1 g of dry extract contained 5225.5 ± 84.9 µmol of Trolox equivalent. This extract showed the highest antioxidant activity followed by *Leontopodium nivale alpinum* (3522.3 ± 91.4), *Helichrysum stoechas* (618.8 ± 13.4 μmol Trolox equivalent/g (dw)), *Medicago sativa* (450.3 ± 13.1 μmol Trolox equivalent/g (dw)), *Plantago lanceolata* (362.2 ± 9.9 μmol Trolox equivalent/g (dw)), *Capsella bursa-pastoris* (360.5 ± 9.2 μmol Trolox equivalent/g (dw)) and finally *Plantago major* (255.9 ± 7.0 μmol Trolox equivalent/g (dw)).

### 3.2. Plant Extracts Inhibited ROS Production by Blood Leukocytes

**Figure 1:**
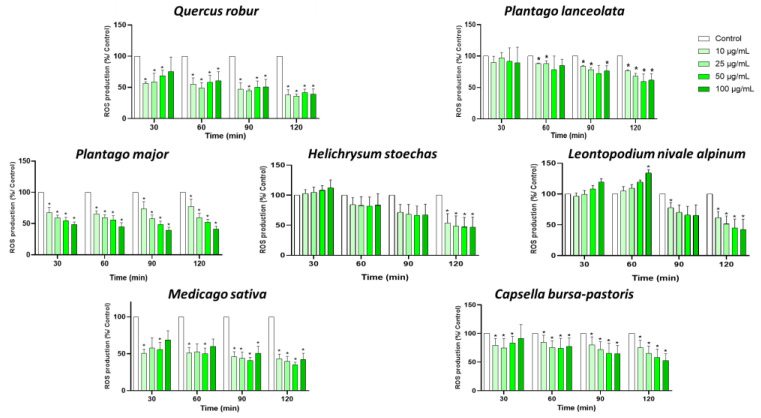
Production of ROS by blood leukocytes with different concentrations of plant extracts. Cells were stimulated with PMA (1 µM) and treated with the indicated concentrations of extracts for 30, 60, 90 and 120 min, and then ROS production was measured. Data are shown as means ± SEM (Control = 100%); * *p* < 0.05 compared with Control.

In order to investigate the antioxidant properties of the seven plant extracts, we evaluated how they affected the formation of ROS in PMA-induced blood leukocytes. After an hour of incubation, PMA stimulation resulted in a notable increase in ROS generation. Treatment with all the extracts inhibited ROS production in a dose-dependent manner (Figure 1), though the reduction was not statistically significant for any of the extracts after 1 h. However, a significant reduction in ROS production was obtained with all plant extracts and at all concentrations after 2 h. At this timepoint and at the concentration of 50 µg/mL, *Medicago sativa* has the most potent effect on ROS production with a reduction of more than 60%, followed by *Quercus robur*, *Leontopodium alpinum* and *Helichrysum stoechas* with 59%, 55% and 53% reduction of ROS production, respectively. *Capsella bursa-pastoris*, *Plantago lanceolata* and *Plantago major* also showed a significant reduction of at least 40%.

**Figure 2:**
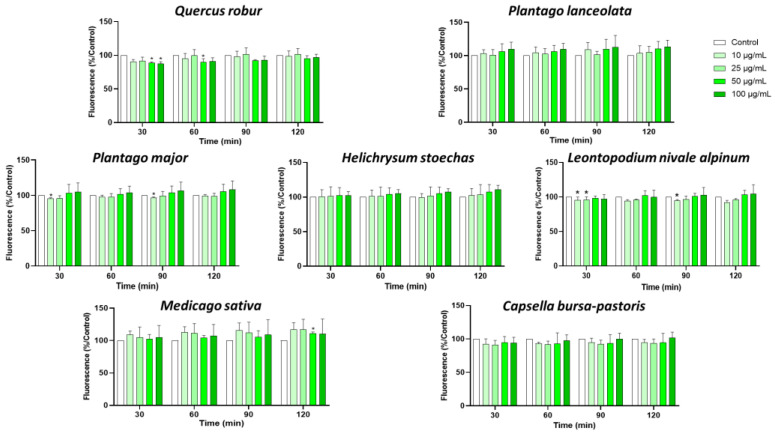
Effect of plant extract on leukocyte viability. Cells were treated with the indicated concentrations of extracts for 2 h, and then cell viability was measured. Data are shown as means ± SEM (Control = 100%); * *p* < 0.05 compared with Control.

These effects were not due to a reduction in cell viability, as the viability assay revealed no significant difference between cells treated or not with plant extracts in the 10–100 µg/mL concentration range after 2 h (Figure 2). In light of these findings, a concentration of 50 µg/mL was chosen for all plant extracts in order to conduct further study.

### 3.3. Plant Extracts Impacted PBMC Cytokine Secretions

**Figure 3:**
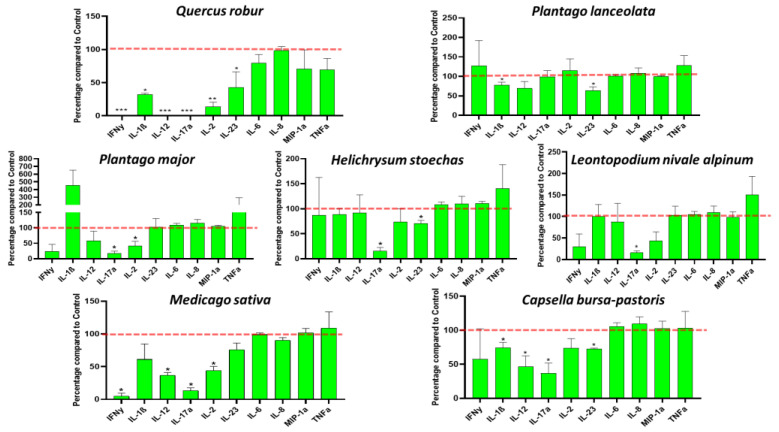
Impact of plant extracts on PBMC cytokine secretions: Cells were incubated with or without PHA (5 µg/mL) and plant extracts (0 or 50 µg/mL) for 24 h. Cytokine levels were measured with Luminex Bio-Plex 200 System. Data are shown as mean ± SEM (Control = 100% indicated by the red line) and analyzed using one-way ANOVA followed by Dunett’s post-hoc test (n = 3). * *p* < 0.05, ** *p* < 0.01, *** *p* < 0.001 compared with Control.

Total PBMCs were stimulated with PHA, either with or without the addition of 50 µg/mL of each extract, in order to evaluate the effects of the seven plant extracts on the inflammatory response. ProcartaPlex^TM^ Immunoassays were then used to measure the concentrations of ten cytokines released into the supernatant (Figure 3). *Leontopodium nivale alpinum* extract seems to have the lowest efficiency, with only IL-17a (−84%, *p* < 0.05) significantly decreased in the supernatant, followed by *Plantago major*, *Plantago lanceolata* and *Helichrysum stoechas* with a significant decrease in two cytokines by each extract (IL-17a (−83%) and IL-2 (−72%), IL-1β (−23%) and IL-23 (−37%), IL-17a (−84%) and IL-23 (−30%), respectively). *Medicago sativa* and *Capsella bursa-pastoris* seemed to be more potent and both of them significantly decreased the secretion of four pro-inflammatory cytokines as follows: IFNγ (−95%), IL-12 (−63%), IL-17a (−86%), IL-2 (−56%) for *Medicago sativa* and IL-1β (−26%), IL-12 (−53%), IL-17a (−63%) and IL-23 (−27%) for *Capsella bursa-pastoris*. Interestingly, the extract from *Quercus robur* exhibited a strong anti-inflammatory impact by reducing the production of six pro-inflammatory cytokines. When stimulated PBMCs were cultured with the *Quercus robur* extract, their secretion of IFNγ, IL-17a, and IL-12 decreased by more than 95% (*p* < 0.001), followed by IL-2 (−86%, *p* < 0.01), IL-1β (−68%, *p* < 0.05) and IL-23 (−58%, *p* < 0.05), compared to non-treated PBMCs. Due to interindividual variations, the *Quercus robur* extract was unable to achieve significance in terms of TNFα, IL-6 and MIP-1a production, despite a downward trend.

### 3.4. Quercus robur Characterization

**Figure 4:**
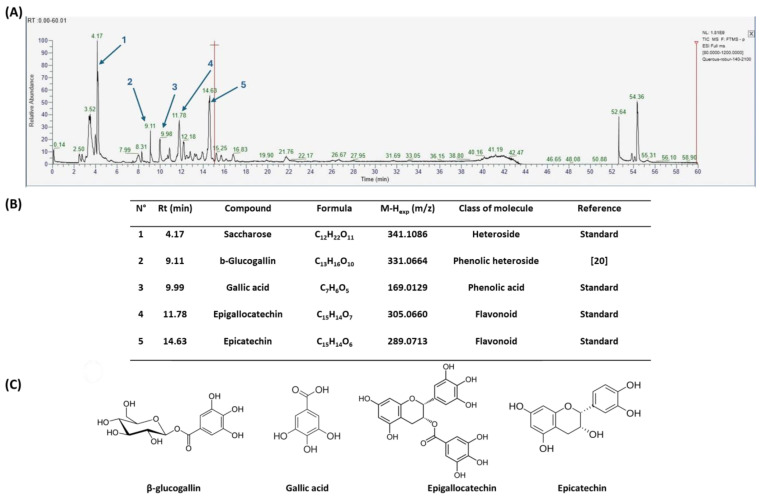
*Quercus robur* extract characterization: (**A**) Chromatogram with compounds identified (numbers 1 to 5 in the chromatogram represent the compounds identified in (**B**)). (**B**) Compounds identified in the *Quercus robur* extract (comparison with analytical standard or according to literature data [20]). (**C**) Chemical structure of the main identified compounds.

Since the *Quercus robur* extract showed the highest inhibition in the cytokine secretion assay, we decided to better investigate its potential anti-inflammatory effect on different inflammatory cells and pathways. Firstly, UHPLC-MS analysis was carried out to better explore the composition of our extract, which led to the identification of β-glucogallin, gallic acid, epigallocatechin and epicatechin as the main compounds (Figure 4).

### 3.5. Quercus robur Inhibited Macrophages Polarization Toward M1-Type

**Figure 5:**
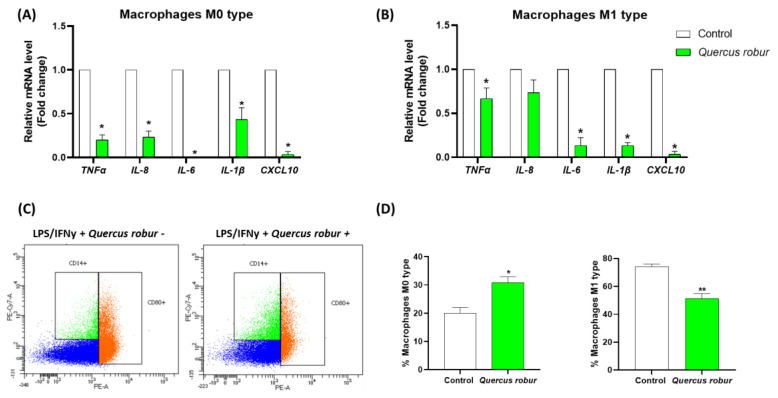
Effect of the *Quercus robur* extract on macrophage polarization: THP-1 cells were supplemented with PMA to allow their activation and then treated with LPS and IFNγ for the polarization into M1-type. The effect of the *Quercus robur* extract (50 µg/mL) was evaluated on (**A**) M0-type macrophages and (**B**) M1-type macrophages. Gene expression was quantified by real-time PCR and normalized using β-actin as an internal control. (**C**) Macrophages were analyzed by flow cytometry. The representative side scatter area plots are shown for CD14 (M0) and CD80 (M1) labeling (Blue represent cells that are negative for both markers, green represent cells that are CD14^+^/CD80^−^ and orange represent cells that are positive for both CD14 and CD80). (**D**) Percentage of M0 (CD14^+^) and M1 (CD14^+^ CD80^+^ and CD14^−^ CD80^+^) macrophages in treated and untreated cells using the total number of labelled cells as the total number of cells. Data are expressed as mean ± SEM and * *p* < 0.05 and ** *p* < 0.01compared with Control.

First, we evaluated how the extract (50 µg/mL) affected the production of pro-inflammatory cytokines in M1-like macrophages. For this, THP-1 cells were stimulated into M0-like, and then IFNγ and LPS were added to polarize them into M1-type. The effective polarization of monocytes into M1-polarized macrophages was confirmed by the increased mRNA expression of M1 macrophage markers, including *IL-1β*, *TNFα*, *IL-6*, *IL-8* and *CXCL10*. Interestingly, the *Quercus robur* extract was able to significantly decrease the expression of all pro-inflammatory cytokines in M0 macrophages (Figure 5A). Additionally, the *Quercus robur* extract attenuated the macrophage response to M1 pro-inflammatory activation through the significant reduction in IL-1β, TNFα, IL-6 and CXCL10 pro-inflammatory cytokines (Figure 5B).

These results were then validated by a flow cytometer assay where M0 and M1 cell surface markers were assessed. *Quercus robur* significantly decreased the percentage of cells expressing the CD80 marker, which reflected the decrease of M1-like macrophages, and significantly increased the percentage of cells expressing only the CD14 marker, reflecting the M0-like macrophages percentage (Figure 5C, 5D). Overall, our results showed that *Quercus robur* inhibited M1 pro-inflammatory activation.

### 3.6. Quercus robur Modulated Several Inflammatory Pathways: Cyclo-Oxygenase 2 (COX-2), STAT-3 and NLRP3 Pathways in LPS-Stimulated Blood Leukocytes

**Figure 6:**
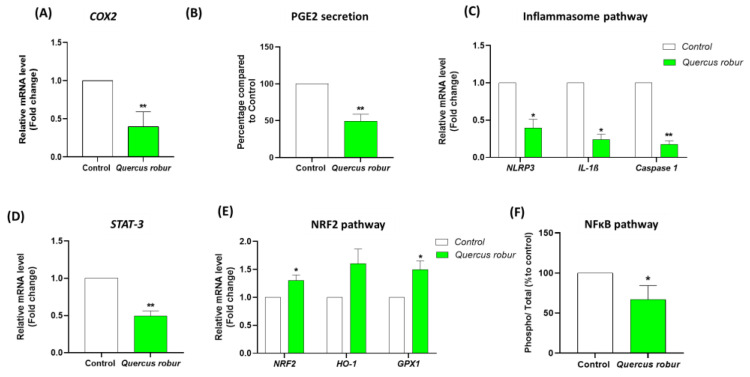
Effect of *Quercus robur* on several inflammatory pathways and antioxidant response in blood leukocytes and PBMCs. Blood leukocytes were incubated with or without LPS (10 µg/mL) and *Quercus robur* (0 or 50 µg/mL). The expression level of different genes was quantified by real-time PCR and normalized using β-actin as an internal control. (**A**) Expression of COX-2 gene. (**B**) Secretion of PGE2 in supernatant compared to untreated cells. (**C**) Expression of inflammasome effector genes. (**D**) Expression of STAT-3 gene. (**E**) Expression of antioxidant-sensitive genes. (**F**) Effect of *Quercus robur* on p65 phosphorylation in PBMCs. Cells were incubated with or without LPS (10 µg/mL) and the extract (50 µg/mL) for 2 h. Pellets from treated cells were collected and NFκB phosphorylation was measured using the NFκB p65 (Total/Phospho) Human InstantOne™ ELISA kit. Data are expressed as mean ± SEM (Control = 100%) (n = 3). * *p* < 0.05, ** *p* < 0.01.

Among the different inflammatory pathways, we explored the COX-2 pathway, an enzyme expressed in response to inflammatory stimuli, leading to the production of prostaglandins, which are hormone-like substances that contribute to the inflammatory response and various physiological processes in the body. It is apparent that *Quercus robur* significantly decreased the expression level of *COX-2* at 50 µg/mL in LPS-stimulated blood leukocytes (Figure 6A). Additionally, the treatment with 50 µg/mL of *Quercus robur* significantly decreased PGE2 secretion in the same cell type (Figure 6B).

Another important inflammatory pathway is the inflammasome pathway mediated by NLRP3. For this, the gene expression of *NLRP3*, *IL-1β*, *IL-18* and *Caspase-1* was measured. As expected, the *Quercus robur* extract strongly decreased the expression of all studied genes, i.e., *NRLP3*, *IL-1B*, *IL-18* and *Caspase-1* (Figure 6C), in LPS stimulated leucocytes.

Finally, since IL-6 activates the JAK/STAT3 pathway, we investigated the effect of the *Quercus robur* extract on *STAT-3* gene expression in the same cell type and we found that the extract significantly decreased *STAT-3* expression in LPS-stimulated cells (Figure 6D).

### 3.7. Quercus robur Modulated NRF2 Pathway

Following the important antioxidant activity of the extract, reflected by a significant decrease in ROS production, we assessed how *Quercus robur* affected the expression of the antioxidant enzymes Heme oxygenase-1 (*HO-1*) and Glutathione Peroxidase 1 (*GPX1*), as well as the transcription factor NRF2. The results showed that the treatment with Quercus robur extract significantly increased the expression of *NRF2* and *GPX1* with no effect on *HO-1* expression (vs stimulated untreated cells) (Figure 6E).

### 3.8. Quercus robur Inhibited NFκB Activation

Finally, since we demonstrated that the *Quercus robur* extract suppressed the expression and production of a number of pro-inflammatory cytokines, we decided to evaluate if this inhibition is mediated by the NFκB pathway. For that, the effect of the *Quercus robur* extract on p65 phosphorylation was assessed in LPS-stimulated PBMCs. As demonstrated in Figure 6F, phospho-NFκB p65 levels were significantly reduced in the PBMCs in the presence of 50 µg/mL *Quercus Robur* extract (vs.untreated controls).

## 4. Discussion

Among the various health benefits documented for natural plant products, the anti-inflammatory effect is of particular interest and stands out as one of the most frequently reported effects [21]. Since ancient times, people tend to use plant products, their parts or extracts to prevent or to treat different acute and chronic illnesses, especially those associated with inflammation. This use was not well-documented or proved until recently, and mainly during the last three decades where an enormous number of studies were published regarding the beneficial effect and medical uses of natural molecules from plants. In recent years, natural molecules from plants are considered as a crucial source in the drug discovery and development process, mainly because plant-derived compounds often have pleiotropic effects, meaning they can modulate multiple biological pathways, making them attractive candidates for drug discovery, repurposing and combination therapies to treat various diseases [22].

The pathophysiology of numerous chronic and autoimmune diseases, such as obesity, diabetes, rheumatoid arthritis, cardiovascular diseases, Alzheimer’s and others, is significantly influenced by chronic inflammation. It is implicated in the causality and severity of these diseases, and understanding the events that occur during chronic inflammation may be the key in discovering new therapeutic agents and treatment approaches leading to its resolution [23].

Here we investigated the antioxidant and anti-inflammatory effects of seven plants in order to propose a new promising extract with pharmacological potential. All of our plant extracts, except *Leontopodium nivale alpinum*, are listed in the ‘Plants list’ released under the Regulation (EU) Nº 2015/2283 and the ‘BelFrIt list’, meaning that these plant substances and preparations are allowed in food supplements in the European Union [24]. Even though *Leontopodium nivale alpinum* is not listed in the BelFrIt, it can be found in some dietary supplements but less commonly [25]. The choice of this plant was due to its potential benefits in skin diseases including dermatitis. It is important to note that these plants have a history of use in traditional medicine for specific health concerns, and some of them were used for inflammation, but scientific evidence is limited, hindering our understanding of potential effects. All of the tested plants proved antioxidant activity, reflected by a significant decrease in ROS production, which was expected due to their rich composition in flavonoids and polyphenols. Regarding the anti-inflammatory activity, more or less pronounced effects have been found and *Quercus robur* was ultimately selected.

Plant-derived medicines often contain complex mixtures of bioactive compounds that can act synergistically or on multiple targets within biological pathways. This multi-target approach may offer advantages over single-target drugs, particularly in treating complex diseases. *Quercus robur* has been subject to extensive research due to its rich composition, since it contains more than 20 active compounds belonging to phenolic acids, flavones and flavonol aglycones and glycosides in addition to a few tannins that contributed to its potential medicinal properties and therapeutic effects. It is well known that *Quercus robur* is the only source of hydrolysable tannins, i.e., ellagitannines which are roburins (A, B, C, D and E) and grandinin. UHPLC-MS analysis of our extract led to the identification of β-glucogallin, gallic acid, epigallocatechin and epicatechin as the main compounds, in addition to a minor percentage of tannins, i.e., vescalagine and castalagine, and this may be attributed mainly to the solvent used during the extraction process.

One of the most significant natural products obtained from plants, β-glucogallin, is a polyphenolic ester and is thought to be the main metabolite in the biosynthesis of hydrolyzable tannins. It is reported to possess a plethora of pharmacological activities including antioxidant and anti-inflammatory properties [26]. β-glucogallin was shown to reduce ROS production and pro-inflammatory cytokine production, and also inhibit the activation of the inflammatory pathways NFκB and NLRP3 [27]. Recently, gallic acid has received increasing attention due to its powerful anti-inflammatory properties that mainly affect the MAPK and NFκB signaling pathways [28]. Thus, it decreases the release of inflammatory cytokines, lowering the inflammatory response. Epicatechins were used in several clinical trials and have shown to mostly affect nitric oxide metabolism and protect from metabolic disorders through the regulation of Nrf2 [29]. Epigallocatechin, the main polyphenol in green tea, exhibits many types of anti-inflammatory, antioxidant and tissue-protective capabilities which may help treat various diseases such as metabolic disorders and cancer. Epigallocatechin has been reported to decrease ROS production and reduce the expression of the NLRP3 inflammasome and its related activation of IL-18, IL-1β and caspase-1 in mice [30].

In our study, we can attribute the anti-inflammatory activity of the *Quercus robur* extract mainly to the inhibition of NFκB activity, that can lead to an important decrease in the production and expression of several pro-inflammatory cytokines. In addition to the activation of the expression of several cytokines, NFκB could induce the expression of NLRP3, serving as a NLRP3 priming signal, which is crucial for inflammasome activation and regulation. The inflammasome is an important pathway in several inflammatory diseases, and its inhibition is considered an effective treatment [31]. Under normal circumstances, the basal expression of NLRP3 is not sufficient to activate the inflammasome pathway, and an activation signal, that could be exposure to LPS, will lead to an increased activation of NFκB and subsequently NLRP3 expression. In our study, we showed that the treatment *Quercus robus* extract partially inhibits NFκB activation by decreasing phospho-P65 production, leading to a decrease in NLRP3 expression and consequently an inhibition of the inflammasome inflammatory pathway [32]. This effect on both pathways can be attributed to β-glucogallin and epigallocatechin, as previously demonstrated by Khan et al. and Mokra et al. [27,30]. Here we demonstrated, for the first time to our knowledge, an inhibitory effect of the *Quercus robus* extract on the inflammasome inflammatory pathway through decreased expression of *NLRP3*, *IL-1β*, *IL-18* and the *caspase 1*.

The decrease of arachidonic acid (AA) metabolism is also seen after *Quercus robur* treatment, reflected by a significant decrease in *COX-2* expression along with a decrease in PGE2 secretion. Elevated COX-2 expression has been implicated in various pathologic conditions, including cancer and inflammation [33]. This effect could be attributed to epigallocatechin, since it has been demonstrated to inhibit COX-2 at both the mRNA and protein levels by Tajamul et al. [34].

On the other hand, antioxidant activity is very important and tightly related to inflammation. In our study, we demonstrated that the *Quercus robur* extract activates the expression of *NRF2* and thus could be considered an activation signal leading to antioxidant activity. NRF2 is a key transcription factor, activated in response to ROS, that regulates the expression of antioxidant enzymes, such as HO-1 and GPX1, which protect against oxidative stress and tissue damage [35]. We demonstrated a significant decrease in the expression of these two enzymes and this effect could mainly be attributed to epigallocatechin, since Hua you et al. showed that treatment with epigallocatechin activated NRF2 and its downstream antioxidant enzyme HO-1 in an adult rat model [36].

Based on our results, it is evident that the *Quercus robur* extract exhibits significant anti-inflammatory and antioxidant activities. It is important to emphasize that these findings are based on in vitro studies. Translating these effects to humans requires careful consideration of several factors, among which bioavailability, pharmacokinetics and the specific tissue distribution of the extract are key determinants. Since in vitro concentrations are generally higher than those achieved in vivo, additional calculations are required to estimate the corresponding human dose. Typically, translation methods that help in estimating the ‘human equivalent dose’ rely on animal models to bridge the gap between in vitro data and human applications, as these models provide insight into factors such as body weight, surface area and other specific factors that account for variations in volume between animals and human physiology, which are essential for dose adjustments [37]. In vitro and in vivo studies evaluating the effects of *Quercus robur* on animal models are limited. However, in a clinical study, an extract derived from oak wood was orally administered and this treatment showed an increase in plasma polyphenol concentrations and enhanced plasma antioxidant capacity after a few days of supplementation [38]. These findings suggest that the *Quercus robur* extract and its active compounds are effectively absorbed, distributed, and utilized by the body. Three additional clinical trials using the same *Quercus robur* extract demonstrated that supplementation at a dose of 300 mg/day resulted in an overall improvement in fatigue symptoms and a significant reduction in oxidative stress. Thus, this dose could serve as a reasonable starting point for future dose translation in our study [39,40,41].

Our results clearly suggest *Quercus robur* as a successful and safe strategy to treat chronic inflammatory conditions, or prevent inflammatory diseases, includeing cardiovascular diseases, diabetes, rheumatoid arthritis, obesity, Alzheimer’s and other autoimmune diseases. To the best of our knowledge, there have been no previously published studies evaluating the effect of *Quercus robur* on several inflammatory pathways (summarized in the graphical abstract) and on polarized macrophages. Nonetheless, there is still a considerable amount of work ahead, including optimizing dosage, administration methods, and the safety profile of concentrated extracts. In conclusion, our study confirmed that the *Quercus robur* extract could emerge as a promising candidate for both treating and preventing inflammation and associated diseases, thereby promoting overall well-being.

## Figures and Tables

**Table 1:** Molecules of interest in the studied plants.

Species	Molecule of Interest	Chemical Structure
*Leontopodium nivale alpinum*	Leontopodic acid A	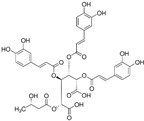
*Quercus robur*	Vescalagine/Castalagine	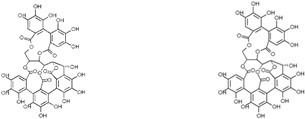
*Medicago sativa*	Tricin	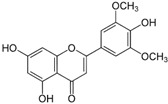
*Helichrysum stoechas*	3,5-dicaffeoylquinic acid	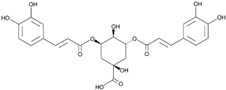
*Plantago major*	Aucubin/verbascoside	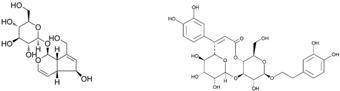
*Plantago lanceolata*	Aucubin/verbascoside	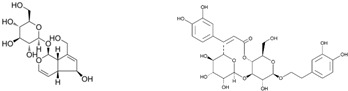
*Capsella bursa-pastoris*	Sulforaphan	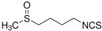

**Table 2:** PCR primer sequences.

Gene	Species	Forward Primer Sequence (5′-3′)	Reverse Primer Sequence (5′-3′)
*ϐ-actin*	Human	CCTGGCACCCAGCACAAT	GCCGATCCACACGGAGTACT
*IL-8*	Human	CTGGCCGTGGCTCTCTTG	CCTTGGCAAAACTGCACCTT
*IL-1* *ϐ*	Human	CCTGTCCTGCGTGTTGAAAGA	GGGAACTGGGCAGACTCAAA
*IL-6*	Human	GCTGCAGGCACAGAACCA	ACTCCTTAAAGCTGCGCAGAA
*TNF* *α*	Human	TCTTCTCGAACCCCGAGTGA	GGAGCTGCCCCTCAGCTT
*CXCL10*	Human	GGAAATCGTGCGTGACATTA	AGGAAGGAAGGCTGGAAGAG
*COX2*	Human	CCCAGGGCTCAAACATGATG	TCGCTTATGATCTGTCTTGAAAAACT
*NLRP3*	Human	CCACAAGATCGTGAGAAAACCC	CGGTCCTATGTGCTCGTCA
*Caspase1*	Human	GCCTGTTCCTGTGATGTGGAG	TGCCCACAGACATTCATACAGTTC
*STAT3*	Human	GCTGCTTAGACGTGGATTT	TAACGTTGAGGGGCATCG
*NRF2*	Human	CACATCCAGTCAGAAACCAGTGG	GGAATGTCTGCGCCAAAAGCTG
*HO-1*	Human	ACAGTTGCTGTAGGGCTTTA	CTCTGAAGTTTAGGCCATTG
*GPX-1*	Human	GCACCCTCTCTTCGCCTTC	TCAGGCTCGATGTCAATGGTC

**Table 3:** Quantification of molecules of interest by HPLC-UV performed using UV with commercial standards.

Species	Molecule of Interest	%g/g of Dry Extract
*Leontopodium nivale alpinum*	Leontopodic acid A	3.77%
*Quercus robur*	Vescalagine/Castalagine	0.03%/0.07%
*Medicago sativa*	Tricin	0.01%
*Helichrysum stoechas*	3,5-dicaffeoylquinic acid	3.24%
*Plantago major*	Aucubin/verbascoside	0.09%/4.51%
*Plantago lanceolata*	Aucubin/verbascoside	0.5%/3.85%
*Capsella bursa-pastoris*	Sulforaphan	/

**Table 4:** Ferric-Reducing Antioxidant Power (FRAP) Assay for plant extracts’ antioxidant activity. Trolox equivalent (μmol) per gram of dry extract was used to compute FRAP.

Species	Trolox Equivalent (µmol/g of Dry Extract)	SD
*Helichrysum stoechas*	618.8	13.4
*Leontopodium nivale alpinum*	3522.3	91.4
*Plantago major*	255.9	7.0
*Plantago lanceolata*	362.2	9.9
*Medicago sativa*	450.3	13.1
*Capsella bursa-pastoris*	360.5	9.2
*Quercus robur*	5225.5	84.9

## Data Availability

The data presented in this study are available on request from the corresponding author.
